# An umbrella review of meta-analyses on the low-FODMAP diet in IBS

**DOI:** 10.3389/fnut.2025.1714281

**Published:** 2026-01-02

**Authors:** Dagmara Bogdanowska-Charkiewicz, Urszula Malinowska, Jarosław Daniluk

**Affiliations:** 1 Department of Gastroenterology and Internal Medicine, Medical University of Bialystok, Bialystok, Poland; 2 Malinowska Studio Urszula Malinowska, Warsaw, Poland

**Keywords:** umbrella review, low FODMAP diet, irritable bowel syndrome, IBS, FODMAP

## Abstract

This umbrella review systematically evaluated the effects of the low FODMAP diet on irritable bowel syndrome (IBS) through 192 studies retrieved from PubMed, Web of Science, Cochrane Library, and Scopus up to January 2025. All meta-analyses and evaluation criteria adhered to PRISMA guidelines. The quality of the included meta-analyses was evaluated by AMSTAR-2. The effect size was expressed as a standardized mean difference, odds ratio, or relative risk, as available. Pooled analysis was based on a random-effects model. Sixteen meta-analyses qualified for the final statistical analysis (141 studies included, 9,904 patients), all of which concerned patients with IBS. Analysis of evidence showed that the low FODMAP diet in IBS patients significantly reduced symptom scores on the IBS Symptom Severity Scale (IBS-SSS) (standardized mean difference (SMD) = −0.599, 5 meta-analyses, 3,761 patients) and improved quality of life (SMD = 0.259, *p* < 0.0001, 5 meta-analyses, 3,576 patients). No significant effect was found on abdominal pain, stool consistency, stool frequency, or microbiota. For bloating, the pooled analysis was not possible due to different measures in the source meta-analyses. The placebo effect was not taken into account in most of the meta-analyses included in the umbrella review. A low FODMAP diet reduces symptoms and improves quality of life in patients with IBS. The results should be approached with caution, as they may be influenced by psychological factors related to the observation itself. As blinding or placebo-controlled conditions are inherently impossible in dietary interventions of this type, it is impossible to decide whether symptom reduction is caused strictly by diet or by non-specific or expectancy-related effects. Further methodologically reliable studies on the effectiveness of the low FODMAP diet in IBS are still needed.

## Introduction

Irritable bowel syndrome (IBS) is a chronic disease of the large and small intestines that is not caused by biochemical or organic changes. This syndrome belongs to the group of gut–brain axis disorders (formerly known as functional disorders). The precise etiology of IBS is unknown (1, 2).

The prevalence of irritable bowel syndrome (IBS) in Europe and North America, estimated based on population studies, is approximately 10–15%. In a meta-analysis of eight international studies, the overall prevalence of IBS was estimated at 11%, according to Rome IV criteria, with significant variation depending on the geographical region. The prevalence of IBS is higher in women than in men (14 and 9%, respectively) (3, 4). The main symptom of IBS is abdominal pain described as a feeling of cramping of varying intensity and periodic exacerbations. It may be constant or recurrent, most often in the lower abdomen and left iliac fossa. In addition, patients often report bloating, and accompanying symptoms of IBS include drowsiness, headaches, back pain, and urinary disorders. Approximately 70% of patients with IBS experience depressive or anxiety disorder symptoms.

Irritable bowel syndrome is diagnosed based on the Rome IV criteria (5). The Rome IV criteria for irritable bowel syndrome (IBS) require recurrent abdominal pain on average for at least 1 day per week in the last 3 months, associated with two or more of the following: the pain is related to defecation; the pain is associated with a change in stool frequency; or the pain is associated with a change in stool form (appearance). The criteria specify that these symptoms must have been present for the last 3 months, with symptom onset at least 6 months prior to diagnosis.

Treatment of IBS includes non-pharmacological and pharmacological treatment. It is recommended to start treatment with non-pharmacological methods.

Due to the fact that the symptoms are chronic in nature, it is important to establish good cooperation with the patient, reassure them that there are no signs of serious disease, explain the chronic nature of the symptoms, and the role of diet, stress, and infections in potentially exacerbating the symptoms. The next step is to educate the patient on how to avoid situations that exacerbate symptoms, modify their diet, and, only if there is no improvement, provide pharmacological treatment (4, 6).

Previous studies have shown the effectiveness of the low fermentable oligosaccharides, disaccharides, monosaccharides, and polyols (FODMAP, LFD) diet in relieving IBS-related symptoms (7, 8). Due to the fact that the research results were ambiguous and varied in terms of methodology, we decided to use an umbrella review to systematize them. In our review, we analyzed meta-analyses on the effectiveness of the low FODMAP diet in reducing symptoms, but it is worth mentioning that the placebo effect was not ruled out, as blinding or placebo-controlled conditions are impossible in the case of interventions such as the low FODMAP diet.

## Methods

### Literature search strategy

Our research comprehensively evaluated the effects of the low FODMAP diet in irritable bowel syndrome. All meta-analyses and evaluation criteria adhered to PRISMA guidelines.

This umbrella meta-analysis utilized data from five online databases: PubMed (https://pubmed.ncbi.nlm.nih.gov/), Cochrane Library (https://www.cochranelibrary.com/), Web of Science (http://isiknowledge.com/), Embase (https://www.embase.com/), and Scopus (http://www.scopus.com/). Our team searched these databases using keywords such as “low FODMAP diet,” “FODMAP,” “irritable bowel syndrome” (IBS), systematic review,” and “meta-analysis”.

### Inclusion criteria and exclusion criteria

Inclusion criteria:

1. Article must be a meta-analysis or a systematic review involving meta-analyses;
2. Articles must describe the low FODMAP diet treatment in IBS;
3. Trials involved adult patients diagnosed according to Rome III or IV criteria (for IBS)

Exclusion criteria:

1. Reviews and systematic reviews without meta-analysis.
2. Animal or *in vitro* studies.
3. Studies involving participants with multiple diseases other than IBS.

Authors excluded abstracts, review articles, observational studies, and case series.

### Data extraction

First, the studies were screened manually by the authors (DBCh and JD).

Second, the full texts of the included studies were carefully reviewed. Third, the authors.

(DBCh and JD) collected pertinent information. The methodological assessment of the studies included in the umbrella review was performed independently by three authors (DBCh, UM, and JD). The statistical analyses were performed by UM.

Baseline and outcome data were collected and saved in Microsoft Excel (Version 16.91). EndNote 21 software was used to eliminate duplicates and substandard documents.

The data extracted included basic literature information (first author name, country, year of publication, funding source, and research registration agreement); experimental details (database used, search date, number of patients in experimental and control groups, intervention type, and duration); and results.

The umbrella review only included the results of studies that evaluated the effectiveness of the low FODMAP diet in IBS. The results of studies that evaluated the effectiveness of the low FODMAP diet in other gastrointestinal diseases were not included in the analysis (9, 10). Studies comparing the effectiveness of the low FODMAP diet in IBS to diets other than normal (e.g., gluten-free diet) were also not included in the umbrella review.

In one case, the effect of the low FODMAP diet concerned IBS symptoms during the inflammatory bowel disease (IBD) remission phase (11), and we assumed an assessment of the diet effect on IBS, excluding other diseases.

### Quality assessment of documentary evidence

The AMSTAR2 tool was used to assess the quality of each included article. AMSTAR2 used 16 questions to assess study design, literature search, literature screening, and data analysis, categorizing articles into four quality levels: high, moderate, low, and critically low (12).

### Statistical analysis

For source meta-analyses that reported pooled effect sizes (standardized mean difference (SMD), mean deviation (MD), odds ratio (OR), or relative risk (RR)) with 95% confidence intervals but without corresponding *p*-values, authors calculated two-tailed p-values using standard statistical procedures. For continuous outcomes (MD, SMD), the standard error was estimated from the confidence interval, and a z-statistic was used to derive the p-value. For binary outcomes (OR, RR), calculations were performed on the logarithmic scale (13).

In order to standardize continuous outcomes across included meta-analyses, all effect sizes were converted to standardized mean difference (SMD). This standardization allowed for the pooling of continuous outcomes measured on different scales, improving the comparability and consistency of the umbrella meta-analysis.

For studies that reported mean difference (MD), these values were transformed into SMD by dividing the MD by the pooled standard deviation of the outcome. When group-specific standard deviations and sample sizes were available, they were used to estimate the pooled standard deviation (13, 14).

Effect sizes reported as Cohen’s d were adjusted using Hedges’ g, a bias-corrected form of SMD that accounts for small sample sizes (15).

In this umbrella meta-analysis, eligible meta-analyses reported binary effect sizes either as odds ratio (OR) or relative risk (RR). In an effort to harmonize effect measures and permit pooled analyses across outcomes, authors initially aimed to convert RRs to OR using established statistical formulas. However, the necessary primary data (e.g., incidence in intervention and control groups) required for the accurate conversion were not consistently reported in the original meta-analyses or their source studies. Despite attempts to obtain the missing data by contacting corresponding authors, no additional information was provided (16–18). Consequently, the authors conducted two parallel sets of quantitative syntheses: one included only meta-analyses reporting OR and the other limited to those reporting RRs. This approach was adopted to preserve the integrity of the reported effect measures and to avoid introducing bias through imprecise conversions.

Statistical analysis was carried out using R 4.2.1 statistical software (R Core Team (2022). R: Language and environment for statistical computing by the R Foundation for Statistical Computing, Vienna, Austria). Packages metafor and forestplot were used. Effect sizes were calculated, including a 95% confidence interval for each outcome variable. A random effects model approach was applied. Visual presentation of the results included a tabularized summary as well as forest plots. Sensitivity analysis (leave-one-out analysis) was conducted to assess the stability of results. Heterogeneity was considered low if I^2^ was ≤ 50% and high if I^2^ was > 50%.

## Results

### Document inclusion process

**Figure 1:**
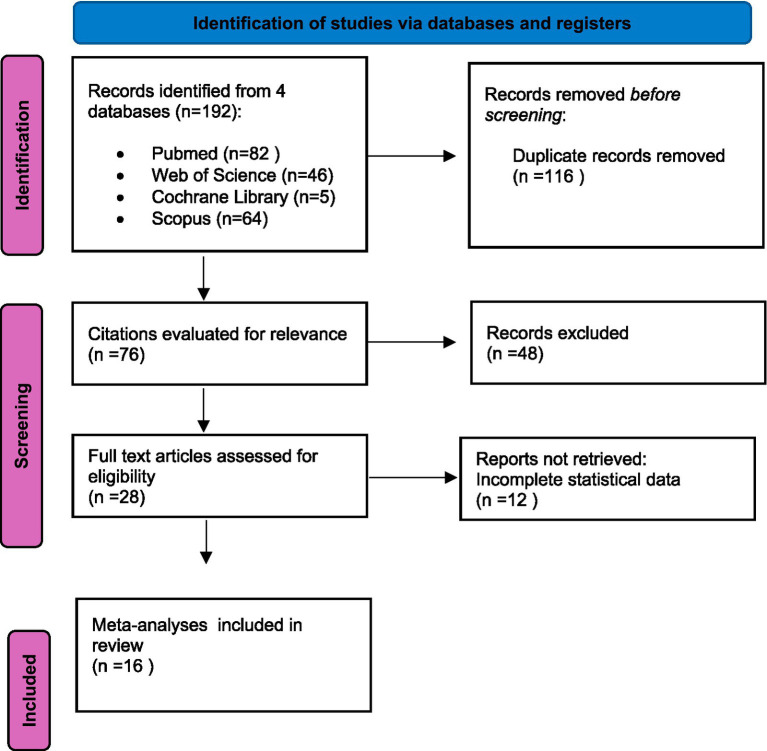
PRISMA flow chart of the document selection process (41).

A total of 290 articles were retrieved from four databases: PubMed, Web of Science, Cochrane Library, and Scopus. After removing duplicates in the first stage, 101 articles remained. Following a thorough review of summaries and titles, 73 articles were excluded. The remaining articles were further screened and classified, resulting in 16 articles being included in this umbrella meta-analysis. The detailed document inclusion process is illustrated in the flow chart (Figure 1).

### Characteristics of the included studies and quality of literature

A total of 16 articles were incorporated into our umbrella analysis, with their detailed characteristics outlined in Table 1. The methodological quality of the included studies was assessed by two independent researchers (DBCh and JD) using the Cochrane risk of bias tool (19).

**Table 1:** Characteristics of selected studies.

First author, year	Intervention type	Outcomes	Disease no	studies included no	patients (total)	AMSTAR 2
Altobelli E., 2017 (16)	Low FODMAP diet	Abdominal pain Bloating Stool consistency Stool frequency	IBS	3	208	High
Black C., 2022 (8)	Low FODMAP diet	Bloating	IBS	13	944	High
Chu P., 2025 (23)	Low FODMAP diet	Microbiota	IBS	4	259	High
Haghbin H., 2024 (27)	Low FODMAP diet	Stool consistency Stool frequency Symptoms	IBS	4	330	High
Hahn J., 2021 (25)	Low FODMAP diet	QoL Stool consistency Symptoms	IBS	22	1,374	High
Jent S., 2023 (20)	Low FODMAP diet	Abdominal pain QoL Stool frequency	IBS	9	604	High
Khan Z., 2025 (26)	Low FODMAP diet	QoL Symptoms	IBS	5	320	High
Lei Y.,2025 (30)	Low FODMAP diet	Symptoms	IBS	7	525	High
Marsh A., 2016 (17)	Low FODMAP diet	Symptoms	IBS	6	354	High
Schumann D., 2017 (21)	Low FODMAP diet	Abdominal pain QoL Symptoms	IBS	9	596	High
So D., 2022 (24)	Low FODMAP diet	Microbiota	IBS	9	403	High
van Lanen A. S., 2021 (1)	Low FODMAP diet	Symptoms	IBS	12	772	High
Wang J., 2021 (29)	Low FODMAP diet	Symptoms	IBS	10	551	High
Xie C., 2022 (28)	Low FODMAP diet	Symptoms	IBS	6	508	High
Yu S. J., 2022 (18)	Low FODMAP diet	Symptoms	IBS	14	1,603	High
Zeraattalab-Motlagh S., 2025 (22)	Low FODMAP diet	Abdominal pain QoL Stool consistency Stool frequency Symptoms	IBS	8	553	High

In all the studies included in meta-analyses, Patient, Intervention, Comparison, and Outcome (PICO) (a framework for developing focused, answerable questions in evidence-based health care) criteria were met. The AMSTAR 2 evaluation revealed that all articles were of high quality.

In all studies, the effectiveness of the low FODMAP diet for IBS was assessed against a normal diet.

#### Direct meta-analysis

The results of meta-analyses are presented in Table 2.

**Table 2:** Results of meta-analyses of different outcomes.

Outcome	Effect size measure	Effect size level	95% CI lower	95% CI upper	*p*	I^2^ (%)	p for heterogeneity	Number of studies included	Number of patients included
Abdominal pain	SMD	−0.130	−0.636	0.376	0.614	93.58	<0.0001	3	1753
	OR	0.44	0.26	0.79	0.006	–	–	1	208
Microbiota	SMD	−0.052	−0.368	0.265	0.749	51.1	0.153	2	662
QoL	SMD	0.259	0.143	0.374	<0.0001	0	0.945	5	3,576
Stool consistency	SMD	−0.240	−0.629	0.149	0.226	87.68	<0.0001	4	2,346
Stool frequency	SMD	−0.126	−0.646	0.393	0.634	90.0	<0.0001	4	1,578
Bloating	RR	0.71	0.47	1.06	0.820	–	–	1	944
	OR	0.32	0.15	0.66	<0.0001	–	–	1	208
Symptoms	SMD	−0.599	−0.708	−0.489	<0.0001	0	0.457	5	3,761
	RR	1.53	1.19	1.97	0.001	65.2	0.035	4	2,420
	OR	0.38	0.23	0.63	0.0002	3.6	0.309	2	1957

CI, confidence interval; OR, odds ratio; SMD, standardized mean difference; RR, relative risk.

##### Abdominal pain

**Figure 2:**
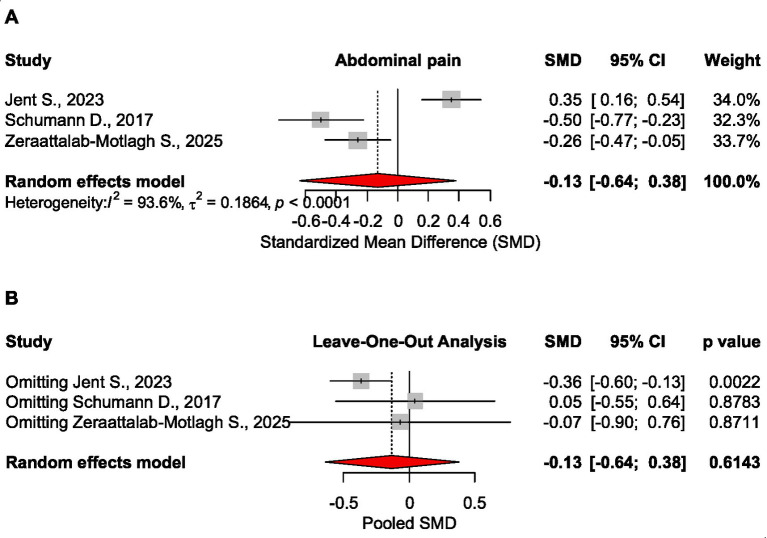
Forest plot **(A)** and sensitivity analysis **(B)** for the effect size of the low FODMAP diet on abdominal pain.

The effect size of the low FODMAP diet on abdominal pain levels was statistically not significant, SMD = −0.130 CI_95_ [−0.636 to 0.376], *p* = 0.614, as based on three meta-analyses, 1753 patients (20–22). Based on sensitivity analysis, when removing Jent S (20) study, the effect size became significant, SMD = −0.363 CI_95_ [−0.596 to −0.130], *p* = 0.002 (Figure 2).

Analysis based on data for frequency of abdominal pain (16) confirmed that patients with a low FODMAP diet had statistically significant lower pain compared to those receiving a traditional diet, OR = 0.44, CI_95_ [0.26 to 0.79], *p* = 0.006 (Table 2).

##### Microbiota

**Figure 3:**
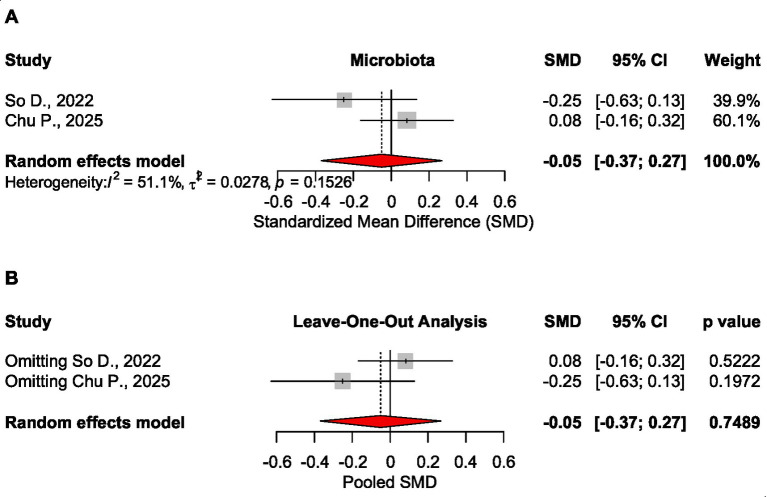
Forest plot **(A)** and sensitivity analysis **(B)** for the effect size of the low FODMAP diet on microbiota.

Analysis confirmed no significant effect of low FODMAP diet on microbiota, SMD = −0.052 CI_95_ [−0.368 to 0.265], *p* = 0.749, 2 meta-analyses, 662 patients (23, 24). Sensitivity analysis showed the stability of this analysis (Figure 3).

##### QOL

**Figure 4:**
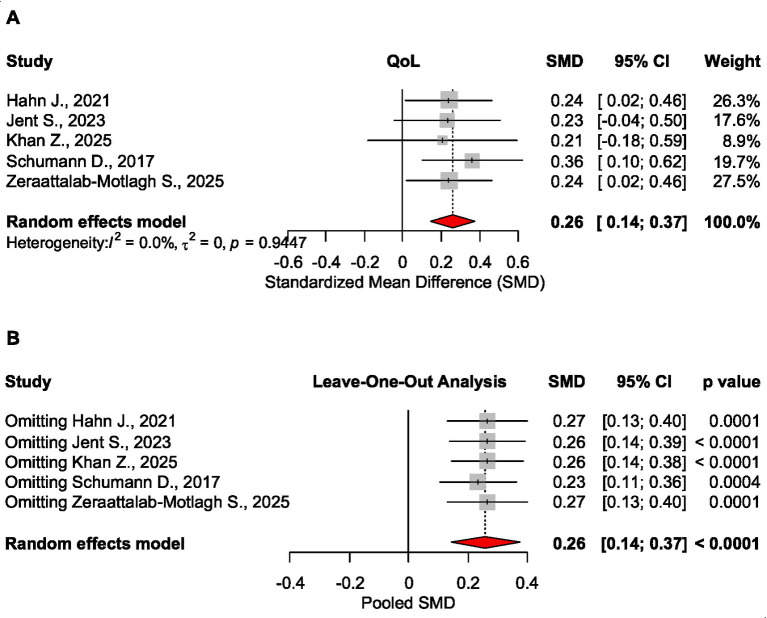
Forest plot **(A)** and sensitivity analysis **(B)** for the effect size of the low FODMAP diet on QoL.

Low FODMAP diet had a significant effect on QoL, SMD = 0.259 CI_95_ [0.143 to 0.374], *p* < 0.0001, 5 meta-analyses, 3,576 patients (20–22, 25, 26). Sensitivity analysis confirmed the robustness of these findings (Figure 4).

##### Stool consistency

**Figure 5:**
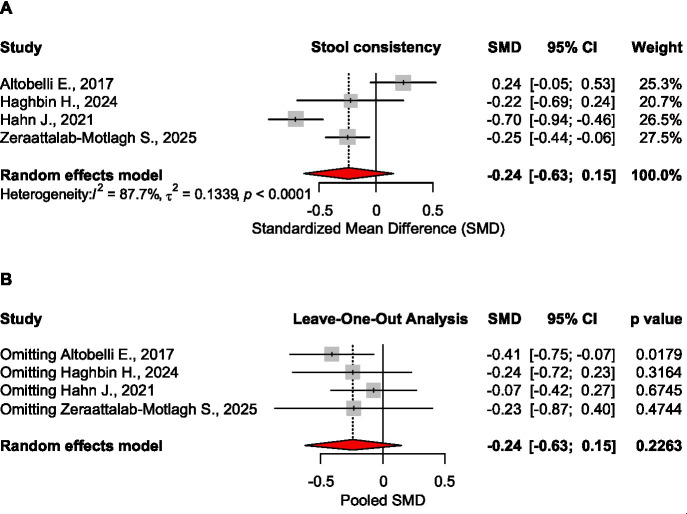
Forest plot **(A)** and sensitivity analysis **(B)** for the effect size of the low FODMAP diet on stool consistency.

Four studies indicate that low FODMAP had no significant effect on stool consistency, SMD = −0.240, CI_95_ [−0.629 to 0.149], *p* = 0.226, 4 meta-analyses, 2,346 patients (16, 22, 25, 27). However, when excluding Altobelli E., (16) study, the effect size was confirmed as statistically significant, SMD = −0.409, CI_95_[−0.747 to −0,070], *p* = 0.018 (Figure 5).

##### Stool frequency

**Figure 6:**
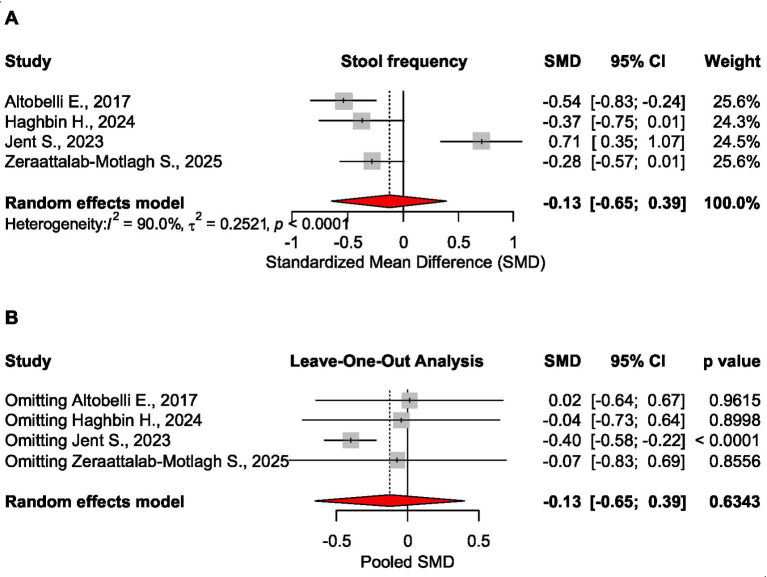
Forest plot **(A)** and sensitivity analysis **(B)** for the effect size of the low FODMAP diet on stool frequency.

Based on 4 meta-analyses, the low FODMAP diet also had no significant effect on stool frequency, SMD = −0.126 CI_95_ [−0.646 to 0.393], *p* = 0.634, 4 meta-analyses, 1,578 patients (16, 20, 22, 27). Exclusion of Jent S, (20) study from the analysis resulted in statistically significant findings, SMD = −0.400 CI_95_ [−0.581 to −0.218], *p* < 0.0001 (Figure 6).

##### Bloating

For bloating, the pooled analysis was not possible due to different measures in source meta-analyses (1 meta-analysis with OR data, 1 meta-analysis with RR data). Based on one study, odds for bloating in patients with low FODMAP were significantly lower vs. the traditional diet, OR = 0.32 CI_95_ [0.15 to 0.66], *p* < 0.001, 208 patients (16). However, based on another study, there was no significant difference in bloating in low FODMAP, RR = 0.71 CI_95_ [0.47 to 1.06], *p* = 0.820, 944 patients (8) (Table 2).

##### Overall symptoms

For symptoms, the data were collected from 11 meta-analyses: 5 having effect size measured with SMD, 4 studies with data in RR, and 2 studies with data in OR.

**Figure 7:**
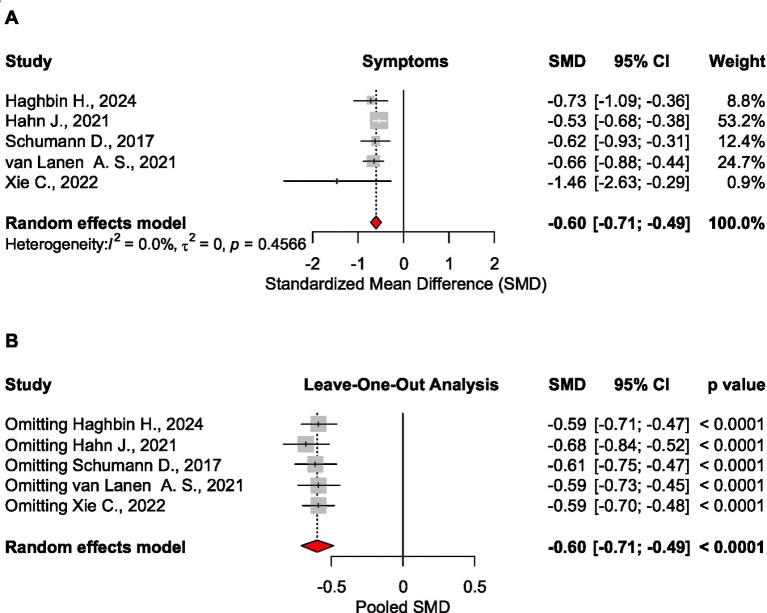
Forest plot **(A)** and sensitivity analysis **(B)** for the effect size of the low FODMAP diet on symptoms based on the standardized mean difference measure.

Analysis including five meta-analyses confirmed a statistically significant effect size of the low FODMAP diet on the symptom level, SMD = −0.599 CI_95_ [−0.708 to −0.489], *p* < 0.001, 3,761 patients (1, 21, 25, 27, 28). The results were confirmed by sensitivity analysis (Figure 7).

**Figure 8:**
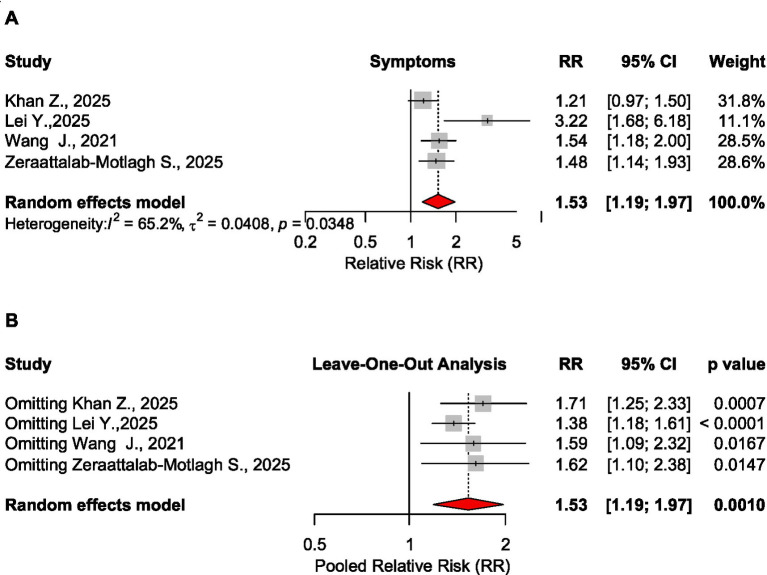
Forest plot **(A)** and sensitivity analysis **(B)** for the effect size of the low FODMAP diet on symptoms based on the relative risk measure.

Based on another 4 meta-analyses, risk for symptom improvement was significantly higher for the low FODMAP diet, RR = 1.53, CI_95_ [1.19 to 1.97], *p* = 0.001, 2,420 patients (22, 26, 29, 30). Sensitivity analysis showed the stability of this analysis (Figure 8).

**Figure 9:**
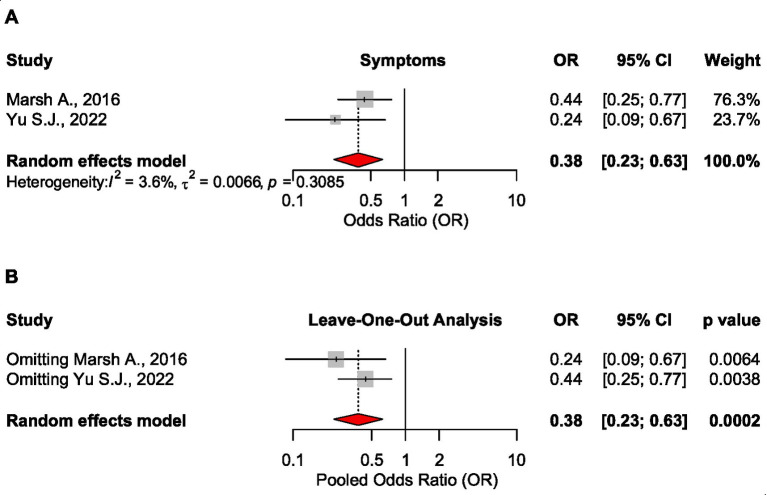
Forest plot **(A)** and sensitivity analysis **(B)** for the effect size of the low FODMAP diet on symptoms based on the odds ratio measure.

The remaining two meta-analyses showed lower odds for symptom improvement in low FODMAP patients as compared to the traditional diet, OR = 0.38 CI_95_ [0.23 to 0.63], *p* < 0.001, 1957 patients (17, 18). Sensitivity analysis confirmed the robustness of these findings (Figure 9).

## Discussion

### Key findings

This comprehensive analysis shows that the low FODMAP diet has beneficial effects in IBS.

The processing of evidence showed that the low FODMAP diet in IBS patients significantly reduced symptom scores on the IBS-SSS scale (SMD = −0.599, 5 meta-analyses, 3,761 patients) and improved quality of life (SMD = 0.259, *p* < 0.0001, 5 meta-analyses, 3,576 patients) (Table 2).

It should be noted that observed improvement, particularly regarding quality of life and the Irritable Bowel Syndrome Symptom Severity Scale, is likely attributable to non-specific treatment effects such as the Hawthorne effect (participants modify an aspect of their behavior in response to their awareness of being observed) or the placebo effect. The last two questions on the IBS-SSS assess the patient’s subjective perception of their condition. Improved scores during the FODMAP diet may thus reflect a change in self-perception rather than an actual improvement in objective symptoms. Unfortunately, blinding or placebo-controlled conditions are inherently impossible in dietary interventions of this type, which is a significant methodological limitation that introduces uncertainty in the interpretation of the observed effects. At the same time, no statistically significant improvement was found in measurable and less perception-dependent symptoms, such as stool frequency or stool consistency, as mentioned below.

The results show improvements mainly in the IBS-SSS and quality-of-life measures. While these outcomes are clinically meaningful, they also include components that are influenced by patients’ perception and psychological factors. In contrast, parameters that are less perception-dependent, such as stool frequency, stool consistency, or microbiota composition, did not show significant differences. This discrepancy should be explicitly discussed as it raises the possibility that part of the reported benefit may reflect non-specific or expectancy-related effects.

No significant effect was found on abdominal pain, stool consistency, stool frequency, or microbiota. For bloating, the pooled analysis was not possible due to different measures in the source meta-analyses. Based on sensitivity analysis (leave-one-out), the results were robust for microbiota, quality of life, and overall symptoms. In case of abdominal pain and stool frequency, the results became significant when removing Jent S., (20) study, while for stool consistency, the effect size was confirmed as statistically significant when excluding Altobelli E., (16) study.

#### Comparison with existing literature

According to the American College of Gastroenterology (ASG) Clinical Guideline, the low-FODMAP diet is the most evidence-based diet intervention for IBS. It consists of three phases: restriction (lasting no more than 4–6 weeks), reintroduction of FODMAP foods, and personalization based on results from reintroduction.

The mechanism of the low FODMAP diet is a reduction in small intestinal absorption of osmotically active short-chain carbohydrates, resulting in diminished intestinal water content and downstream effects on colonic fermentation and gas production (16, 31, 32). Studies have suggested that the low FODMAP diet also reduces the serum levels of proinflammatory interleukins and the levels of fecal bacteria (*Bifidobacterium*, *Faecalibacterium prausnitzii, and Actinobacteria*) (33). The response to a low-FODMAP diet may be associated with factors related to patient demographics, microbiome composition and metabolism, and IBS subtype (34, 35). The clinical response to a low FODMAP diet may be related to different subtypes of IBS, demographic differences, or differences in the composition of the patients’ microbiome.

A diet low in FODMAPs is recommended for patients diagnosed with IBS after a trial of traditional diet modifications, including soluble fiber supplementation and avoidance of gas-producing foods (36).

Our results regarding symptom reduction are consistent with the existing literature (6, 21, 25, 27).

Surprisingly, we did not find a statistically significant reduction in pain after applying the diet, but this may be related to the use of different scales for pain intensity.

According to our data, the low FODMAP diet does not significantly affect stool frequency and consistency, but it should be noted that numerous studies included in the analysis involved patients with both diarrhea and constipation, or mixed types of IBS (7, 36–40).

In case of the analysis of stool habits changing in IBS-D (diarrhea type), Hahn et al. showed a significant decrease in stool frequency compared to the control group (25).

Microbiota results were non-significant, which can be explained by a short intervention duration, different analysis methods, and baseline microbiota diversity.

#### Strengths and limitations

The main strength of this review is that we prepared the first umbrella review on the efficacy of the low FODMAP diet in IBS. Umbrella reviews are reviews of previously published systematic reviews or meta-analyses, and consist of the repetition of the meta-analyses following a uniform approach for all factors to allow their comparison (13). They represent one of the highest levels of evidence synthesis currently available, which increases the credibility of our conclusions. We included 16 high-quality studies, and most studies had a low risk of bias.

A limitation is that most studies included in our review had a different duration of the diet, and most of them were short-term interventions, and they may not reflect long-term symptom control.

The other reason for heterogeneity may be variability in adherence reporting and the absence of data on concurrent pharmacotherapy across included meta-analyses.

Besides, most of the meta-analyses used for the umbrella review did not distinguish between IBS subtypes. Furthermore, the conclusions of the existing analyses did not include conclusions specific to individual IBS subtypes. Therefore, the results concerning the improvement in stool consistency and stool frequency should be interpreted with caution.

The placebo effect and the Hawthorne effect were not analyzed in the meta-analyses used in the umbrella review. These effects may influence the results of the low FODMAP diet in self-reported symptom questionnaires.

#### Clinical implications

In clinical practice, it is recommended to use a dietary intervention in IBS before introducing pharmacotherapy. According to our analysis, the low FODMAP diet significantly reduces these symptoms and improves quality of life, although part of the reported benefit may reflect non-specific or expectancy-related effects caused by the placebo effect.

## Conclusion

This study concludes that low FODMAP in IBS patients reduces symptoms and improves quality of life. At the same time, no statistically significant effect was found on such important symptoms as abdominal pain, bloating, and frequency of bowel movements. While these outcomes are clinically meaningful, they also include components that are influenced by patients’ perception and psychological factors. The results should be approached with caution, as they may be influenced by psychological factors related to the observation itself. Conducting studies that exclude the placebo effect and the Hawthorne effect is unfortunately impossible with dietary interventions such as the low FODMAP diet. Further methodologically reliable studies on the effectiveness of the low FODMAP diet in IBS are still needed.
